# COVID-19 and Molecular Genetics

**DOI:** 10.3390/genes13040676

**Published:** 2022-04-12

**Authors:** Giuseppe Novelli, Michela Biancolella

**Affiliations:** 1 Department of Biomedicine and Prevention, Tor Vergata University of Rome, 00133 Rome, Italy; 2 Department of Biology, Tor Vergata University of Rome, 00133 Rome, Italy

Since early December 2019, the COVID-19 pandemic has impacted global society: over 400 million people have been infected with SARS-CoV-2, and there have been nearly 6 million deaths worldwide (1.4%) (Johns Hopkins Coronavirus Resource Center, CRC, https://coronavirus.jhu.edu/map.html, accessed on 28 February 2022). Added to this are consequences of the long-term effects of SARS-CoV-2 (long COVID Syndrome, LCS) which leads to millions of people suffering from exhaustion, cognitive problems and other long-lasting symptoms after infection [1]. The exact causes of LCS are not still known, but it is certainly a consequence of the infection. It is estimated that at least 10% to 30% of people who are infected with the coronavirus can develop long-term symptoms. It is not clear why some people develop LCS for a long time and others do not. It is interesting to note that this feature is quite common in SARS-CoV-2 infection. In fact, since the first studies on COVID-19, a great heterogeneity in phenotypic expression has emerged with asymptomatic subjects, patients with mild forms of the disease, subjects with severe forms that require hospitalization, and others with a more severe phenotype who end up needing intensive care [2]. Although most exposed individuals become infected, rare individuals have also been observed who do not become infected despite repeated exposure [3]. This considerable inter-individual clinical variability constitutes an as-yet unclear scientific and medical enigma that is not clear [1].

In the past two years, there has been great progress in unraveling this conundrum and thereby contributing to the understanding of pathogenesis of COVID-19 [4]. Numerous studies of human genetics have been produced; some of which published in this Special Issue of *Genes* [5,6,7,8,9,10,11]. Common and rare variants have been identified using traditional human genetics approaches: genome-wide association studies (GWAS) and direct sequencing of genes coding for protein involved in precise biochemical pathways implicated in the pathogenesis of the infection [12,13,14,15]. These studies have made it possible to identify alleles of increased susceptibility and/or partial resistance to the COVID-19 disease, in coding and non-coding regions of genes. However, certainly the most important contribution emerged from the International Consortium COVID Human Genetic Effort (www.COVIDhge.com, accessed on 28 February 2022), which made it possible to find for the first time that about 3% of patients with critical COVID-19 pneumonia had congenital errors of immunity (IEI) that compromise innate and interferon-mediated immunity as a result of mutations in the genes TLR3, TLR7, and IRF7 (especially in patients < 65 years) [16,17]. In addition, the same consortium found the presence of autoantibodies (auto-Abs) neutralizing interferon type I in at least 10% of other patients with severe disease [17]. Overall, this research has shown that 20% of severe COVID-19 cases have a defect in the interferon circuit [18]. This proportion is unprecedented among infectious diseases [15]. However, what is the genetic basis of the remaining 80% of patients? Some of the articles published in this Special Issue have suggested additional candidate genes and identified pathways and study strategies that may receive confirmation in the coming months on different case series and different populations. For example, Mbarek et al. [11] compared whole-genome sequencing data of 14,398 adults and Qatari-national with 925 Italian individuals using an innovative genome approach and identified a subset of genes involved in innate immunity and host–pathogen interactions. Russo et al. [9] examined the coding sequences of 10 common variable immunodeficiency-associated genes obtained by the whole-exome sequencing of 121 hospitalized patients and showed significant enrichment in predicted pathogenic point mutations in severe patients compared with non-severe patients. These data not only confirm and extend the involvement of IEIs in COVID-19, but contribute to building the rationale for individualized management based on B-cell therapy. Monticelli et al. [7] provide evidence that a variant (p.Val197Met) (rs12329760) in the *TMPRSS2* gene has a deleterious effect on protease and a protective effect on the patients. Its role appears particularly relevant in two subgroups of patients—young males and elderly women—and among those affected by co-morbidities, where the variant frequency is higher among individuals who are mildly affected by the disease and do not need hospitalization or oxygen therapy than among those more severely affected, who required oxygen therapy, ventilation, or intubation. This study provides useful information for the identification of patients at risk of developing a severe form of COVID-19, and encourages the usage of drugs affecting the expression of *TMPRSS2* or inhibiting protein activity. Indeed, recent evidence supports the idea of inhibiting TMPRSS2 activity as a possible COVID-19 therapy [7].

It is evident that by broadening the knowledge of human genetics and deepening the studies on innate and adaptive immunity, together with the effects of the different variants of SARS-CoV-2 that have emerged or will emerge, it will be possible to develop new diagnostic tests and personalized medicine protocols.

Overall, the papers in this Special Issue cover various genetic and molecular aspects of SARS-CoV-2 infection, suggesting how integrating biological knowledge of the pathogen and the host may lead to possible new pandemics in the future.
